# Genomic Analysis of Haloarchaea from Diverse Environments, including Permian Halite, Reveals Diversity of Ultraviolet Radiation Survival and DNA Photolyase Gene Variants

**DOI:** 10.3390/microorganisms11030607

**Published:** 2023-02-28

**Authors:** Sagorika Nag, Priya DasSarma, David J. Crowley, Rafael Hamawi, Samantha Tepper, Brian P. Anton, Daniel Guzmán, Shiladitya DasSarma

**Affiliations:** 1 Blue Marble Space Institute of Science, Seattle, WA 98104, USA; 2 Department of Microbiology and Immunology, University of Maryland School of Medicine, Baltimore, MD 21201, USA; 3 Department of Biological and Physical Sciences, Assumption University, Worcester, MA 01609, USA; 4 New England Biolabs, Ipswich, MA 01938, USA; 5 Centro de Biotecnología, Faculty of Sciences and Technology, Universidad Mayor de San Simón, Cochabamba 4962, Bolivia; 6 Institute of Marine and Environmental Technology, University of Maryland System, Baltimore, MD 21202, USA

**Keywords:** radiation, halophile, stratosphere, solar radiation, archaea, extremophiles

## Abstract

Ultraviolet (UV) radiation responses of extremophilic and archaeal microorganisms are of interest from evolutionary, physiological, and astrobiological perspectives. Previous studies determined that the halophilic archaeon, *Halobacterium* sp. NRC-1, which survives in multiple extremes, is highly tolerant of UV radiation. Here, *Halobacterium* sp. NRC-1 UV tolerance was compared to taxonomically diverse Haloarchaea isolated from high-elevation salt flats, surface warm and cold hypersaline lakes, and subsurface Permian halite deposits. *Haloterrigena*/*Natrinema* spp. from subsurface halite deposits were the least tolerant after exposure to photoreactivating light. This finding was attributed to deviation of amino acid residues in key positions in the DNA photolyase enzyme or to the complete absence of the photolyase gene. Several *Halobacterium, Halorubrum* and *Salarchaeum* species from surface environments exposed to high solar irradiance were found to be the most UV tolerant, and *Halorubrum lacusprofundi* from lake sediment was of intermediate character. These results indicate that high UV tolerance is not a uniform character trait of Haloarchaea and is likely reflective of UV exposure experienced in their environment. This is the first report correlating natural UV tolerance to photolyase gene functionality among Haloarchaea and provides insights into their survival in ancient halite deposits and potentially on the surface of Mars.

## 1. Introduction

Halophilic Archaea (Haloarchaea) are well known for their polyextremophilic character, including tolerance not only to high salinity and desiccation, but also to toxic perchlorate salts, anaerobic conditions, and ionizing and ultraviolet radiation. Their recent isolation from 250 million-year-old subsurface halite deposits suggested the possibility of survival over geologic time and a mode for dispersal through space [1,2,3,4]. The discovery of halite deposits on Mars in recent years has even raised the potential for their existence in refugia on the Red Planet [5]. Experiments have shown that some Haloarchaea can survive in Earth’s stratosphere in a metabolically active state with conditions similar to those on the surface of Mars [4,6,7,8,9]. The combination of tolerance to such extremes is extraordinary among microbes on Earth and provides a framework for understanding the limits to life as we know it.

On Earth, Haloarchaea are found in hypersaline environments with highly variable light conditions, from intense sunlight to complete darkness. Many physiological studies have been directed at determining the responses of model haloarchaeal strains, e.g., *Halobacterium* sp. NRC-1 (NRC-1), through genetic and transcriptomic approaches, including its response to a wide range of the electromagnetic spectrum [10,11]. NRC-1 displays remarkable tolerance to UV and serves as a model for potential alien life forms that may survive on the surface of Mars or in the hostile environment of space [4,12,13,14,15]. Comparative genomic studies have suggested that many, if not most Haloarchaea may have gene content coding for these physiological capabilities [16,17,18].

In addition to its UV tolerance, the photoresponse of NRC-1 is remarkable and includes phototrophic, phototactic, and buoyancy capabilities [4,19,20]. Under illuminated conditions, NRC-1 is known to induce production of its purple membrane and gas-filled vesicles, promoting growth especially under microaerobic conditions [21,22]. Bacteriorhodopsin, a retinal protein in the purple membrane, carries out light-driven proton pumping and drives ATP synthesis [23]. Such a simple phototrophic system utilizing the most intense part of the solar spectrum may have evolved prior to the rise of photosynthesis, very early in the history of life on Earth, with the subsequent photosynthetic systems (chlorophyll) evolving to use the adjacent parts of the solar spectrum, as proposed in the Purple Earth hypothesis [3]. Bacteriorhodopsin and other retinal-based pigments also provide new biosignatures for exoplanet research [3,24]. 

Phototrophic growth of *Halobacterium* sp. NRC-1 and the associated flotation to the surface using gas vesicle nanoparticles (GVNPs) in solar salt ponds under intense solar irradiation exposes cells to a significant dose of damaging UV radiation, including potentially UV-C (<290 nm). While UV-C is primarily responsible for triggering the formation of thymine dimers and other photoadducts (e.g., 6-4 photoproducts), it is filtered out by Earth’s ozone layer in the modern era. The lack of UV shielding on Mars means that high doses of damaging radiation continue to reach the planet’s surface similar to the conditions on early Earth [25,26]. The sequencing of the *Halobacterium* sp. NRC-1 genome (the first Haloarchaeon to be fully sequenced) revealed the presence of genes for a wide variety of DNA repair systems, including direct photorepair, nucleotide excision repair, mismatch repair, oxidative repair, non-homologous end-joining, and recombinational repair [10,27,28]. The genome was found to contain two *phr* genes coding for photolyase-like proteins and the *uvr*ABC and D genes that code for DNA excinuclease activity [27,28]. All of these gene transcripts and proteins were identified by transcriptomic and proteome analyses, respectively [13,29].

Both light and dark repair were found to be carried out efficiently in NRC-1, which is more than twice as tolerant to UV-C as yeast, five times more tolerant than *Escherichia coli*, and nearly fifty times more tolerant than human cells [13,14,15]. Genetic analysis of NRC-1 by gene knockout has shown that highly efficient UV damage repair occurs by the direct photorepair system using Phr2 [12]. The related Phr1 protein with 42% identity with Phr2, was not found to be involved with photorepair and is yet of unknown function. Additional genetic knockout studies showed that dark repair in NRC-1 occurs through UvrABCD excinuclease-mediated excision repair, with deletion of *uvr*A, *uvr*C, or a double deletion of the two resulting in greatly diminished dark repair [12,14]. Transcriptomic analysis, however, found no coordinated bacterial-type of SOS system, and only a few genes were induced by UV [15]. 

**Figure 1:**
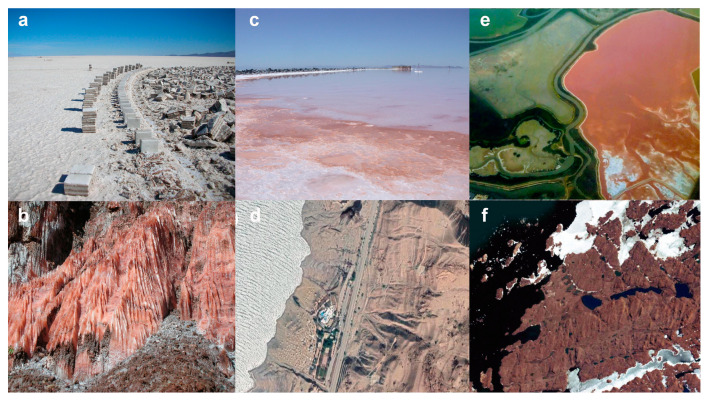
Isolates were obtained from halite deposits from two locations in Bolivia and brine from the Great Salt Lake and Dead Sea. (**a**) Salar de Uyuni in Potosí, Bolivia; (**b**) Sal Rosada (pink salt), Tarija, O’Connor Province, Bolivia; (**c**) Great Salt Lake, North Arm; (**d**) Dead Sea, Jordan; (**e**) Aerial photo of a crystallizer pond from a San Francisco Bay Solar Saltern, California, U.S.A., reproduced with permission from Satyajit DasSarma; (**f**) Deep Lake (rhombus-shaped lake in image), Antarctica. Panels d and f were reproduced with permission from Find Latitude and Longitude, https://www.findlatitudeandlongitude.com (accessed on 13 April 2022).

Although the remarkable tolerance of *Halobacterium* species to UV light has been studied in detail, few comparative studies of UV damage repair have been conducted using diverse and recent environmental isolates of Haloarchaea. Our isolation and sequencing of seven new and diverse Haloarchaea from different geographic locations, including a high-elevation salt flat, 250 million-year-old subsurface Permian halite deposit, perennially cold lake, and high- and low-lying seas made such a study feasible (Figure 1). Consequently, a combination of comparative genomic and experimental studies was carried out using these isolates along with the well-characterized model microbes: the mesophilic NRC-1 and cold-adapted Antarctic *Halorubrum lacusprofundi* (Hla), focusing on UV survival after photorepair [30]. The findings show that a wide range of UV-C tolerance correlates with diverse environmental conditions where they were isolated. Moreover, based on comparison to the published structure of the *Synechococcus elongatus* PCC 6301 enzyme [31,32], UV tolerance was found to be correlated with photolyase gene functionality in the genomes of these Haloarchaea.

## 2. Materials and Methods

### 2.1. Strains

The study included nine sequenced strains (Table 1). Two strains were laboratory models: *Halobacterium* sp. NRC-1 (NRC-1), a model organism isolated from South San Francisco Bay solar salterns, California, USA [4,27,33] and *Halobacterium lacusprofundi* (Hla), isolated from Deep Lake, Antarctica [30,34]. The remaining strains were recent isolates (Figure 1): *Halobacterium* sp. GSL-19 from the North Arm of the Great Salt Lake, UT, USA [35], *Salarchaeum* sp. JOR-1 from the east coast of the Dead Sea, Jordan [36], *Halorubrum* sp. BOL3-1 [37] and *Halobacterium* sp. BOL4-2 from Salar de Uyuni, Bolivia [38], and *Haloterrigena salifodinae* (BOL5-1) [39], *Natrinema versiforme* (BOL5-4), and *Natrinema pallidum* (BOL6-1) from Tarija mines in Bolivia [40]. Haloarchaea were grown at 37 °C, in culture tubes with CM^+^ [41].

### 2.2. UV Survival Assays

Log phase cultures were diluted 1:100 in 2 mL of basal salts (250 g NaCl, 20 g MgSO_4_, 2.0 g KCl, 3.0 g Na-citrate, 2.3 mg FeCl_2_, 440 µg ZnSO_4_, 330 µg MnSO_4_, and 10 µg CuSO_4_ (per 1L)) [41] and placed in glass Petri dishes to a depth of ~1 mm and irradiated with a FG15T8 Ultraviolet Germicidal lamp emitting 254 nm UV-C radiation to the doses of 0, 24, 48, 96 and 144 J/m^2^. Ten-fold serial dilutions were performed in basal salts and 20 microliter samples were spotted in duplicate on CM^+^ plates (CM^+^ plus 20 g/L agar). One unwrapped and one foil-wrapped plate for each dose was exposed to light for two hours using a Philips F32T8 Daylight lamp. All plates were then wrapped in aluminum foil and incubated in airtight containers at 40 °C for 5–14 days before counting colony forming units (CFU).

### 2.3. Phylogenetic Analysis

A local database combining the proteomes of all the organisms of interest was constructed (https://halo-ed.org/halo-gen/web_links.htm, accessed on 1 June 2022) and used to query with protein sequences of interest using BLAST. Protein IDs (PIDs) of top hits were identified and sequences extracted using NCBI Batch Entrez. Phylogenetic analysis including multiple sequence alignments, identity matrices, and neighbor joining phylogenetic trees were constructed using Clustal Omega [42] and Phylogeny.fr [43,44].

### 2.4. Gene Organization

For gene organization analysis, genomic regions around *phr*2 were compared. To address synteny, a genetic map was made using the HaloWeb and KEGG mapping tools for each of the 9 organisms [45,46]. The coding regions were identified, genes were drawn to scale, and color-coded to aid visualization.

### 2.5. Protein Structural Analysis

The crystal structure of the *Synechococcus elongatus* PCC 6301 (previously known as *Anacystis nidulans*) DNA photolyase complex with thymine dimer analog (complex A) and flavin adenine dinucleotide (FAD) and 8-hydroxy-5-deazaflavin (HDF) cofactors (Research Collaboratory for Structural Bioinformatics Protein Data Bank, RCSB PDB: 1TEZ, and SWISS-MODEL Template Library: 1TEZ.1: SMTL Chain Id:B/PDB Chain Id:A) was used as the basis for analysis and homology modeling. First, each individual amino acid of the *S*. *elongatus* (SEL) photolyase model was examined using Swiss-Pdb Viewer to identify residues proximal to DNA, FAD or HDF [47]. In this study, the SWISS-MODEL photolyase numbering, which excludes the initiator methionine, was used.

Next, multiple sequence alignment of the SEL and haloarchaeal photolyase sequences (when present) was performed in order to identify conserved and variant residues. A homology model of the NRC-1 photolyase was constructed using Swiss-Model using the SEL photolyase as the template [47]. The template had a Global Model Quality Estimate (GMQE) score of 0.75 and a QMEANDisCo global score of 0.73 ± 0.05, indicating high coverage and accuracy in template-target alignment along with superior quality of the 3D protein structure. A high structural similarity between the two was estimated by a template model score of about 0.95–0.97. Magic fit option from Swiss-Pdb Viewer was used to superimpose the SEL photolyase model and the homology model of NRC-1 to check for structural similarity. Key amino acid residues in the SEL photolyase were substituted with haloarchaeal residues using Swiss-Pdb Viewer to determine any effect on interactions with HDF, DNA, or FAD.

## 3. Results and Discussion

### 3.1. Phylogeny of Diverse Haloarchaea

**Figure 2:**
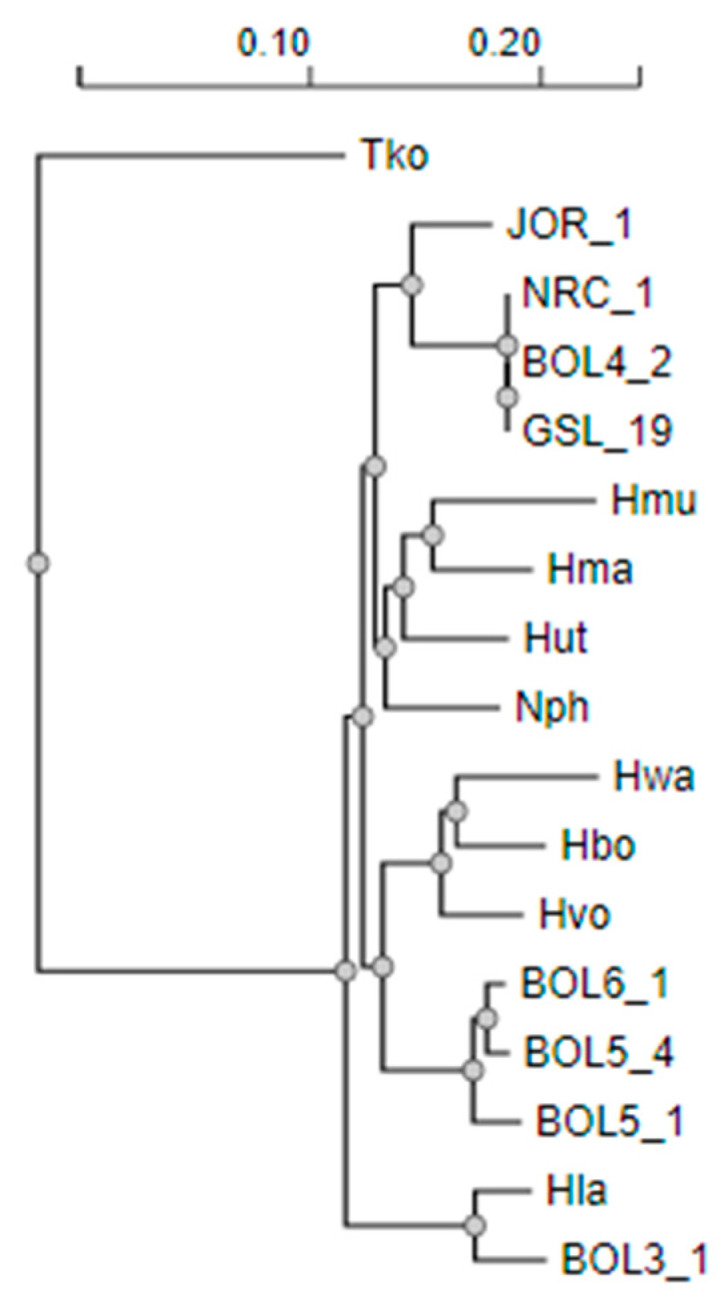
Phylogenetic NJ-tree of 16S rRNA from Haloarchaea. Laboratory models *Halobacterium* sp. NRC-1 and *H. lacusprofundi* (Hla) and newly isolated Bolivian strains *Halorubrum* sp. BOL3-1, *Halobacterium* sp. BOL4-2, *H. salifodinae* BOL5-1, N. *versiforme* BOL5-4, and *N. pallidum* BOL6-1 and *Halobacterium* sp. GSL-19 from the Great Salt Lake and *Salarchaeum* sp. JOR-1 from the Dead Sea. Additional species shown include *Halomicrobium mukohataei* (Hmu), *Haloarcula marismortui* (Hma), *Halorhabdus utahensis* (Hut), *Natronomonas pharaonis* (Nph), *Haloquadratum walsbyi* (Hwa), *Halogeometricum borinquense* (Hbo), *Haloferax volcanii* (Hvo), and *Thermococcus kodakarensis* (Tko). Multiple alignment: MAFFT, Alignment curation: BGME, Tree inference: FastME, Tree rendering: Newick Display [43].

Haloarchaea representing a variety of genera isolated from diverse hypersaline habitats included (a) three strains isolated from brine: *Halobacterium* sp. NRC-1, *Halobacterium* sp. GSL-19, and *Salarchaeum* sp. JOR-1; (b) one isolate from sediment: *Halorubrum lacusprofundi*; (c) three from underground halite: *Haloterrigena salifodinae* BOL5-1, *Natrinema versiforme* BOL5-4 and *Natrinema pallidum* BOL6-1; and (d) two from surface salt crust: *Halorubrum* sp. BOL3-1 and *Halobacterium* sp. BOL4-2. The phylogenetic tree constructed from their 16S rRNA sequences reflected the diversity of Haloarchaea (Figure 2). Clusters of Haloarchaea were apparent, reflecting the three orders, Haloarchaeales with *Halobacterium* spp. and *Salarchaeum* sp., Haloferacales with the *Halorubrum* spp., and Natrialbales with *Haloterrigena* and *Natrinema* spp.

### 3.2. Genomic Characteristics of Diverse Haloarchaea

**Figure 3:**
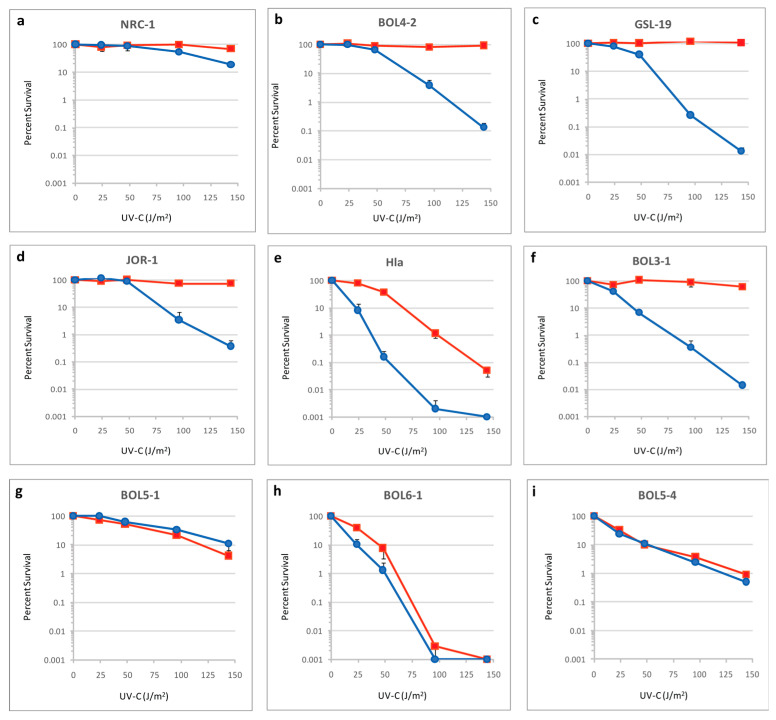
Survival of diverse Haloarchaea after UV irradiation. Shown are plots of percent survival of different strains versus dose of 254 nm wavelength light (24 to 144 Joules per meter squared, J/m^2^), with (red lines) or without (blue lines) exposure to photoreactivating light: (**a**) *Halobacterium* sp. NRC-1; (**b**) *Halobacterium* sp. BOL4-2; (**c**) *Halobacterium* sp. GSL-19; (**d**) *Salarchaeum* sp. JOR-1; (**e**) *Halorubrum lacusprofundi* (Hla); (**f**) *Halorubrum* sp. BOL3-1; (**g**) *Haloterrigena salifodinae* BOL5-1; (**h**) *Natrinema pallidum* BOL6-1; (**i**) *Natrinema versiforme* BOL5-4.

Haloarchaeal cultures in log-phase were tested for survival after irradiation with increasing doses (0, 24, 48, 96 and 144 J/m^2^) of UV-C (254 nm), either with or without exposure to photoreactivating light. The strains exhibited varying degrees of UV survival in the presence and absence of photoreactivating light (Figure 3, red versus blue lines, respectively). The three *Halobacterium* spp. (NRC-1, BOL4-2, and GSL-19) and *Salarchaeum* sp. JOR-1 group exhibited the best light survival characteristics after irradiation, with strain NRC-1 being the most UV tolerant of the group. This group, along with BOL3-1, showed the expected high UV resistance in the presence of photoreactivating light, with the ability for complete survival even after doses as high as 144 J/m^2^. However, with the notable exception of NRC-1, each of the other four strains showed reduced UV tolerance in the absence of photoreactivating light, with three to four logs of killing after the 144 J/m^2^ UV dose.

The two *Halorubrum* spp. showed lower UV tolerance in the absence of light. However, compared to *H. lacusprofundi* (Hla), strain BOL3-1 had considerably better photorepair capability, with survival levels similar to those found in the *Halobacterium*/*Salarchaeum* group. This difference likely reflects the conditions in the environments where the two were isolated, with Hla from light-deprived sediment at the bottom of Deep Lake and BOL3-1 from highly irradiated Salar de Uyuni salt crust. The *Haloterrigena* and *Natrinema* spp. showed little to no light survival capabilities. The lack of photorepair in these three strains is consistent with their dark, subsurface habitat.

### 3.3. Photolyase Genes and Gene Organization

**Figure 4:**
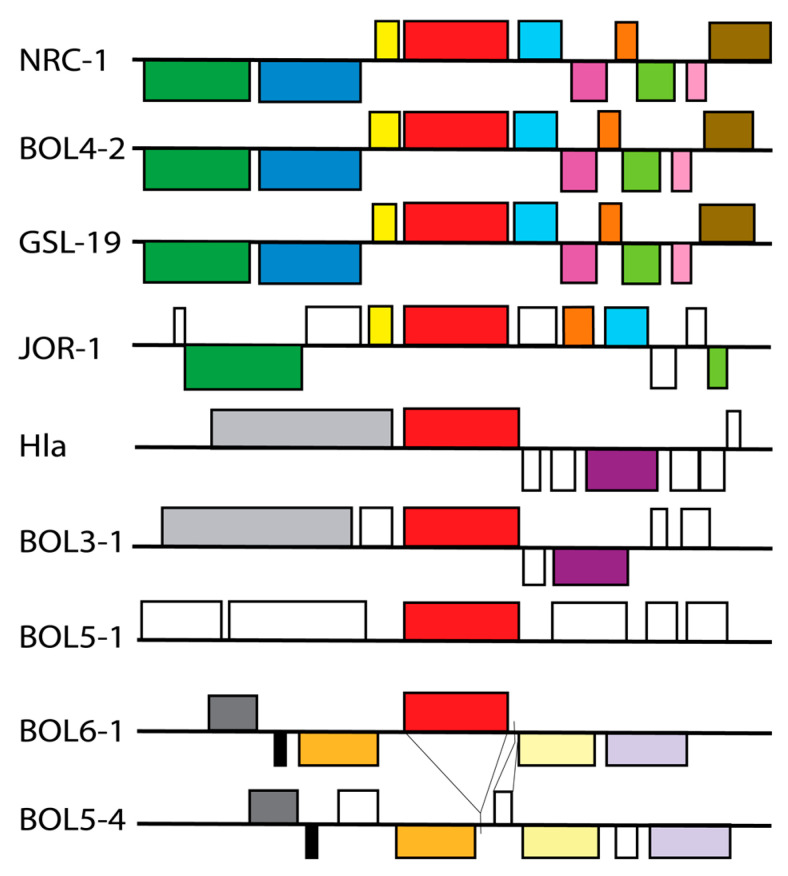
Gene organization around the *phr*2 gene in NRC-1, BOL4-2, GSL-19, JOR-1, Hla, BOL3-1, BOL5-1, and BOL6-1 and corresponding region in BOL5-4. Genes are color-coded with the direction of transcription indicated by placement above (rightward) or below (leftward) thick back lines. The long-chain fatty acid-CoA ligase genes are shown in dark green, sulfatase-like hydrolase/transferase genes in dark blue, thioesterase genes in dark yellow, *phr*2 genes red, *sod*2 genes in light blue, genes corresponding to a hypothetical protein in magenta, DUF5827 family protein genes in dark orange, MBL fold metallo-hydrolase genes in light green, cupin domain-containing protein genes in pink, FAD-dependent thymidylate synthase genes in brown, DEAD/DEAH box helicase genes in light gray, GHMP kinase genes in purple, DUF115 domain-containing protein genes in dark gray, FGF80_09825-like hypothetical genes in black, cell division protein genes in light orange, sugar phosphate isomerase/epimerase genes in light yellow, Gfo/Idh/MocA family oxidoreductase genes in light purple, and other non-homologous genes in white. The deletion and insertion of DNA in the *phr*2 gene region of BOL5-4 are indicated by lines between the BOL5-4 and BOL6-1 maps.

The *phr*2 gene, coding for the functional photolyase of NRC-1 along with the proximal region, was compared in the other Haloarchaea to identify sequence conservation, synteny, and the presence of other genes related to DNA repair. Of the five Haloarchaea exhibiting both superior levels of UV tolerance and photorepair (NRC-1, BOL4-2, GSL-19, JOR-1 and BOL3-1), each contained a highly conserved *phr*2 gene but varied in the extent of synteny in the *phr*2 region (Figure 4). Of the remaining four Haloarchaea with inferior UV survival and photorepair properties, three strains, BOL5-1, BOL6-1, and Hla, contained *phr*2 genes with considerable numbers of sequence differences, and one strain, BOL5-4, lacked the *phr*2 gene entirely (see below). The NRC-1 Phr2 gene product was found to be nearly (99%) identical to that of BOL4-2, GSL-19 and JOR-1, while Hla and BOL3-1 exhibited a similar degree of identity to NRC-1, ~69%, and BOL5-1 and 6-1 were more distant, with 61% and 65% identity, respectively (data not shown).

Gene organization around *phr*2 showed similarities and differences depending on the strain. In the NRC-1 genome, *phr*2 was found to be adjacent to *sod*2 encoding superoxide dismutase, which is known to be involved in DNA repair via protection against oxidative damage (Figure 4) [27]. In transcriptomic analysis, *phr*2 and *sod*2 were found to be transcriptionally linked and co-induced in response to exposure to UV [48,49]. The *Halobacterium* species (NRC-1, BOL4-2 and GSL-19) exhibited synteny around *phr*2 (Figure 4). JOR-1 had a similar arrangement, however, with two genes of unknown function located between *phr*2 and *sod*2.

The more distantly related Haloarchaea showed no synteny with NRC-1 around *phr*2. When comparing the region in the relatively closely related *Halorubrum* (Hla and BOL3-1), and *Haloterrigena* and *Natrinema* genera (BOL5-1, BOL6-1, and BOL5-4), each group exhibited similar gene organization, but they all lacked a *sod* gene or any other repair genes nearby. One of the most interesting findings was that the BOL5-4 genome lacked the *phr*2 gene altogether, with a 1727 bp deletion in the region as well as a 237 bp insertion containing a short open reading frame (FEJ8110390) of unknown function.

### 3.4. Structural Analysis of Photolyases

**Figure 5:**
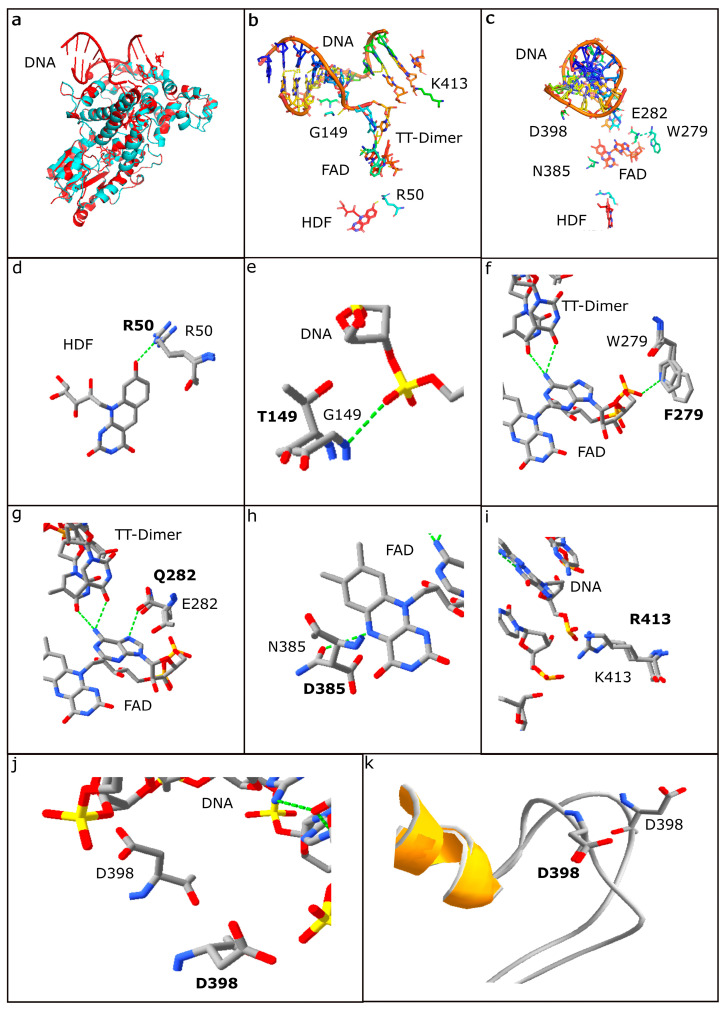
Crystal structure of *S*. *elongatus* PCC 6301 (SEL) photolyase compared to the *Halobacterium* sp. NRC-1 Phr2 model (amino acids shown in bold), including key amino acid positions, visualized using PyMOL (panels (**a**–**c**)) and Swiss-Pdb Viewer (panels (**d**–**k**)). (**a**) The ribbon structure of the SEL photolyase (red) superimposed with the NRC-1 Phr2 model (blue); (**b**) bound DNA, including CPD dimer analog (TT-Dimer), of the SEL photolyase along with the FAD and HDF cofactors interacting with nearby amino acids; (**c**) bound DNA of SEL photolyase rotated by 90 degrees. (**d**) Residue R50 in SEL shown superimposed with corresponding R residue in NRC-1; (**e**) G149 in SEL shown superimposed with T in NRC-1; (**f**) W279 in SEL shown superimposed with F in NRC-1; (**g**) E282 in SEL shown superimposed with Q in NRC-1; (**h**) N385 in SEL shown superimposed with D in NRC-1; (**i**) K413 in SEL shown superimposed with R in NRC-1; D398 in SEL and D in NRC-1 are shown in stick (**j**) and ribbon models (**k**).

For the reference, the model of the crystal structure of *S*. *elongatus* PCC 6301 (SEL) photolyase bound to a synthetic 14-nucleotide double stranded DNA oligomer containing a TT-cyclobutane pyrimidine dimer (CPD) analog was used [31,32]. The structure also included the essential FAD and HDF cofactors required to catalyze photorepair. In order to examine the impact of the differences in photolyases, a homology model of the NRC-1 photolyase was constructed with the SEL enzyme as a template using Swiss-Model (see Materials and Methods). After the two photolyases were found to be structurally nearly identical, the SEL structure was used to replace residues differing in other haloarchaeal photolyases to visualize the impact of substitutions observed in NRC-1 and other Haloarchaea (Figure 5) [31].

Based on studies by Tamada et al. [32] and Mees et al. [31] and our analysis of the SEL photolyase crystal structure, a total of 59 residues were identified that were proximal (within ~2.8 Å) to or interacting with either the bound DNA, or FAD and HDF cofactors. When these residues were compared with corresponding residues in photolyases of Haloarchaea using multiple sequence alignments and structural comparisons, 15 amino acid residues, including six key positions previously reported (R50, G149, E282, N385, D398 and K413) in the SEL photolyase, were found to be changed in at least one of the haloarchaeal photolyase sequences, (see Figure 5b,c) [31,50,51]. 

When the 15 amino acid residues in the SEL photolyase were substituted with corresponding haloarchaeal residues in the model, it was determined that two strains exhibiting inferior light repair, BOL5-1 and BOL6-1, and were found to have many non-conservative substitutions compared to the NRC-1 sequence (Figure 3g,i; Table 2). In the BOL5-1 Phr, a total of 12 substitutions were observed compared to NRC-1, including nine residue differences in amino acids associated with DNA interactions (P144H, V147D, Y148F, T149S, Y150D, Q282R, D398N, R413T, R460H), two residues implicated with FAD interactions (N384H, D385A), and a single residue with HDF interaction (R50V). For BOL6-1, only five differences were found, all in DNA binding residues (T139P, P144H, Y148F, T149S, and R413E) (Table 2). The large numbers of differences were observed in key residues for BOL5-1 and BOL6-1 Phr gene products, correlating with their reduced degree of photorepair capabilities.

Of the two *Halorubrum* spp., Hla, which was isolated from lake sediment, exhibited lower light survival (Figure 3e). This strain exhibited substitutions in three key photolyase residues compared to NRC-1. Two such substitutions, L242M and F279Y, would result in altered FAD-interactions, which may affect photolyase packing and function. The third substitution in Hla compared to NRC-1, the conservative change, T149S, was identified at a residue implicated in DNA interaction and was also identified in the photorepair defective BOL5-1 and BOL6-1 strains. In contrast, *Salarchaeum* sp. JOR-1, isolated from Dead Sea brine, has two substitutions compared to NRC-1, L242V and R413D, which apparently do not diminish its photorepair ability. BOL3-1 and the two NRC-1-like *Halobacterium* spp., BOL4-2 and GSL-19, with much better photorepair and light survival (Figure 3b,c,f), exhibited no substitutions in key residues when compared to NRC-1, reflecting the overall close similarity of their genomes (Table 2).

### 3.5. HDF Binding Residues

**Figure 6:**
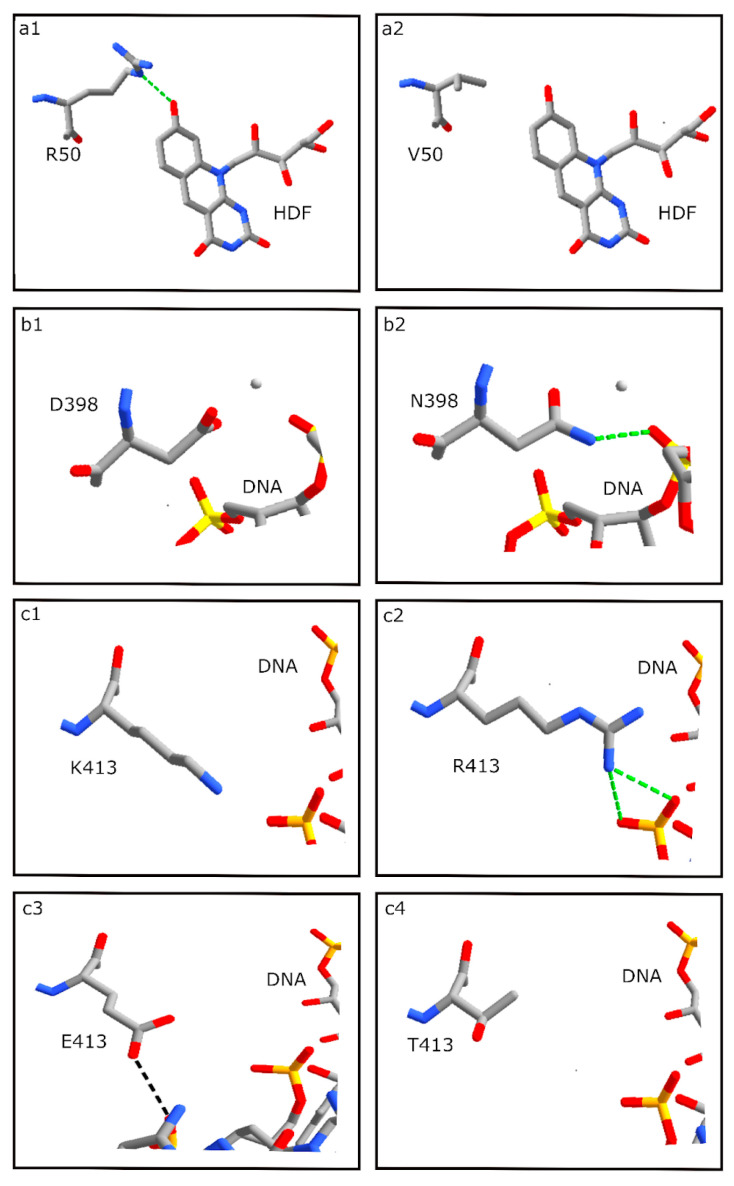
Analysis using Swiss-Pdb Viewer of interactions of natural or substituted amino acids at positions 50, 398 and 413 in the SEL photolyase with HFD cofactor or bound DNA. (**a1**) R50 in SEL photolyase showing an H-bond with HDF; (**a2**) R50V substitution in BOL5-1 showing no H-bond formation between V and HDF; (**b1**) D398 in SEL photolyase shows no H-bond; (**b2**) D398N substitution in BOL5-1 showing H-bond formation between N and DNA. Mg^2+^ ion is shown as a gray sphere; (**c1**) K413 in SEL photolyase showing no H-bond formation with DNA, FAD or HDF; (**c2**) K413R substitution in NRC-1 showing two weaker H-bonds (black dashed lines) formation between R and DNA; (**c3**) K413E substitution in BOL6-1 showing a weak H-bond formation between E and DNA; (**c4**) K413T substitution in BOL5-1 showing no H-bond formation between T and DNA. Green dashed lines indicate stronger H-bonds in panels (**a1**,**b2**,**c2**), and the black dashed line indicates a weaker H-bond in panel (**c3**).

All of the haloarchaeal strains in our study harboring a photolyase gene contained the key HDF binding residue, R50, with one exception, BOL5-1. This strain, which was isolated from subsurface halite, had an R50V substitution that resulted in the loss of a hydrogen bond (H-bond) with HDF, consistent with loss of function and the observed inferior photoreactivation capability (cf. Figure 6a1,a2). Among Haloarchaea, the photolyase of *Halobacterium halobium*, a closely related strain to NRC-1, had been previously identified as a HDF group based on spectroscopic photoreactivation studies [52]. Consistent with this report, the haloarchaeal photolyases examined here appear to be most closely related to *S. elongatus* PCC 6301, which uses HDF, in contrast to the *Escherichia coli* photolyase, which uses 5,10-methenyltetrahydrofolate (MTHF) [32,53,54,55].

### 3.6. DNA Binding Residues

Among the predicted DNA binding residues examined, ten were substituted with non-conserved amino acids in BOL5-1 and BOL6-1, consistent with both strains exhibiting reduced photorepair capability. The SEL D398 residue is substituted with N in BOL5-1 but was conserved in all of the other haloarchaeal photolyases (Figure 6b). While the SEL D398 residue did not form a H-bond to the photolyase-bound DNA, the D398N substitution in BOL5-1 results in a predicted H-bond between the N residue and DNA, suggesting an enhanced effect on DNA binding and potential perturbation of photolyase function. Such an enhanced binding to DNA may be responsible for a deleterious effect on photolyase function and may result in reduced photorepair in BOL5-1.

Another residue of interest, the SEL K413 position (Figure 6c1), which is proximal to but not directly bound to DNA, was substituted with R in the Haloarchaea with good to excellent photorepair, including NRC-1, BOL4-2, GSL-19 and BOL3-1. Two new H-bonds were observed between R and DNA, apparently without deleterious effect due to similar size and positive charge for both amino acid side chains (Figure 6c2). The Haloarchaea with relatively poor light repair ability, in contrast, showed likely deleterious substitutions. In BOL6-1, a K413E substitution resulted in replacement of a basic residue with an acidic residue, and a weak H-bond was predicted between the E residue and slightly different region of the DNA segment (cf. Figure 6c2,c3). In BOL5-1, a K413T substitution of the basic residue with a polar, uncharged residue resulted in loss of H-bonding to DNA (Figure 6c4). Thus, it appears that in the haloarchaeal and *S*. *elongatus* photolyases, the position corresponding to K or R413 likely serves an important role in DNA binding. The difference between the strong H-bond in NRC-1 and similar *Halobacterium* spp. and absence of a H-bond in BOL5-1 or weakened H-bond in BOL6-1 is consistent with the reduced ability of the latter two haloarchaeal subsurface strains to carry out efficient light repair. JOR-1 with good photorepair capability contains a D residue in the same position.

Several additional residues interacting with DNA in the SEL photolyase were found to be altered in haloarchaeal strains with reduced photorepair compared to NRC-1, including V147D, Y150D, N384H, and R460H in BOL5-1, T139P in BOL6-1, and P144H and Y148F in both BOL5-1 and BOL6-1 (Table 2). A majority of the differences resulted in alterations in charge (P144H, V147D, Y150D, and N384H), but in these cases, the changes were not found to result in clearly deleterious effects based on replacement analysis (data not shown). Consequently it is unclear whether these differences are responsible for the observed phenotypic differences. 

### 3.7. FAD Binding Residues

**Figure 7:**
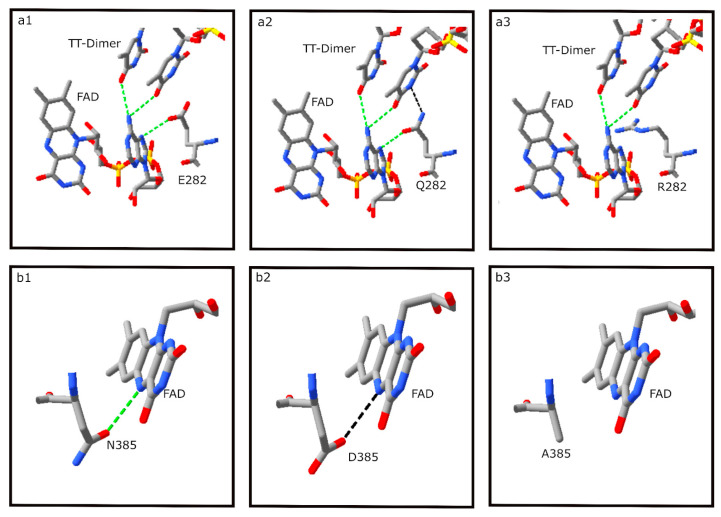
Analysis using Swiss-Pdb Viewer of interactions of natural or substituted amino acids at positions 282 and 385 of SEL photolyase with bound DNA or FAD cofactor. (**a1**) E282 in SEL showing H-bond formation between E and FAD (green dashed lines); (**a2**) E282Q substitution in NRC-1 showing H-bonds (green dashed lines) between Q and FAD, and a weaker H-bond (black dashed line) between Q and the TT-Dimer; (**a3**) E282R substitution in BOL5-1 showing no H-bond formation between R and FAD/TT-Dimer; (**b1**) N385 in SEL showing an H-bond formation between N and FAD (green dashed line); (**b2**) N385D substitution in BOL6-1 showing a weak H-bond (black dashed line) between Q and FAD; (**b3**) substitution N385A in BOL5-1 showing no H-bond formation between R and FAD.

Among the four *S. elongatus* photolyase amino acid residues interacting with the FAD cofactor, two were substituted in BOL5-1 and BOL6-1, and two were substituted in Hla with possible deleterious consequences (Table 2). E282 was substituted with Q in nearly all Haloarchaea, with the exception of the poorly photorepairing strain BOL5-1, where it is substituted with an R (Figure 7a1–a3). While E282 forms an H-bond with FAD, the E282Q replacement in most haloarchaeal organisms forms two H-bonds, one strong and one weak, with the FAD cofactor and TT-Dimer, respectively, and the BOL5-1 E282R replacement results in the loss of interactions between the amino acid and both the FAD cofactor and TT-Dimer.

An additional residue interacting with the FAD cofactor, the SEL N385, is substituted with D in most of the haloarchaeal organisms with the exception of BOL3-1, which contains an N. However, in the poorly photorepairing strain BOL5-1, N385 is substituted with A (cf. Figure 7b1,b2). This results in replacement of a strong H-bond between the amino acid and FAD in SEL with a weak H-bond in most Haloarchaea, while in BOL5-1, substitution with A results in loss of the H-bond (7b3), consistent with a deleterious effect on the BOL5-1 photolyase and the observed poorer photorepair.

### 3.8. Environmental Implications

Since early studies of Haloarchaea established efficient photorepair in *Halobacterium* species, this property was considered to be a general characteristic of extremely halophilic microbes [16,17,56,57]. The rationale was that the hypersaline environments were damaging to DNA due to high solar radiance and periods of desiccation, resulting in the selection for their high GC-composition and genetic variability [58,59]. The present study of diverse Haloarchaea isolated recently from the environment together with two well-studied laboratory strains has established unexpected variability in photorepair and UV tolerance. Genomic analysis coupled with structural comparison indicates that these observed phenotypic differences reflect the occurrence of photolyase variants, and in one case, absence of the gene. Consequently, there is molecular and genomic basis for the correlation between Haloarchaea isolated from the sediment or subsurface halite exhibiting poorer survival and photorepair characteristics and surface brines exhibiting generally excellent UV tolerance. 

Based on observations from the survival curves (Figure 3), it is evident that BOL5-4, BOL5-1, and BOL6-1, and to a lesser degree Hla, which were isolated from the subsurface, lack a robust photoreactivation system (Table 2). For BOL5-4, the phr2 gene is missing entirely, with little to no photorepair. Analysis of other *Natrinema* genomes in NCBI revealed the lack of a photolyase gene in the genome of another isolate from a subsurface mine, *Natrinema* sp. YPL30, suggesting that loss of the *phr*2 gene may be relatively common in subsurface species (GCF_013456555.2). Synteny was nevertheless observed in the most closely related species, e.g., *Natrinema* sp. BOL6-1 and BOL5-4, but not with *Haloterrigena* sp. BOL5-1, which is taxonomically somewhat more distant. BOL6-1, in comparison, showed fewer changes and may have slightly higher functioning of its photorepair system compared to BOL5-1 (Table 2). 

The Antarctic Deep Lake brine sediment isolate *H. lacusprofundi* is a relatively UV-sensitive and poorly photorepairing strain of the *Halorubrum* genus. In comparison, the closely related recent isolate *Halorubrum* sp. BOL3-1 from the high-elevation surface (3659 m) salt crust exhibited a greater degree of tolerance to UV (Figure 3). This likely reflects the lower levels of exposure to damaging UV rays of the former, which is shielded by a deep brine layer, while the latter was isolated from surface salt crust at high elevation that is regularly exposed to high levels of solar radiation. The photorepair function of BOL3-1 is also superior to that of Hla.

Similar to BOL3-1, other highly UV-tolerant Haloarchaea with excellent photorepair systems included BOL4-2, also isolated from the high-elevation surface salt crust, and GSL-19 and JOR-1, which were isolated from brines near the surface of two large hypersaline terminal lakes, Great Salt Lake and Dead Sea. A majority of the amino acids corresponding to important positions in photolyase are conserved across these species (Table 2). The photolyases in the *Halobacterium* spp. are nearly identical to one another, while for JOR-1 and BOL3-1, a few substitutions were observed, but primarily not in the most important residues for enzyme function. 

In addition to photolyase-mediated photorepair, Haloarchaea are known to utilize other repair systems, including both bacterial and eukaryotic types [57]. Among the most important for dark repair is the bacterial-type nucleotide excision repair system (UvrABCD), which is widely distributed among Haloarchaea [16,17]. The Uvr system has been shown to play an important role in the high UV tolerance of NRC-1 through genetic and transcriptomic analysis [14]. Another repair system operating in NRC-1 is a recombinational repair system involved in utilizing the eukaryotic-type RPA-like single-stranded DNA binding protein, which has been shown to be involved in ionizing radiation tolerance [11,60]. It seems likely that a broader study of haloarchaeal repair systems and other extremophilic microorganisms [61] will reveal other interesting genomic differences reflecting the diversity of environmental niches in which they are found.

## 4. Conclusions

Remarkable diversity in UV resistance and photorepair systems was identified among the seven newly isolated Haloarchaea and two well-studied laboratory strains. A range of phenotypes in UV tolerance was observed, with those isolated from the surface brine or salt flats, *Halobacterium* spp. NRC-1, BOL4-2, and GSL-19, *Salarchaeum* sp. JOR-1, and *Halorubrum* sp. BOL3-1 exhibiting higher levels of survival with light repair compared to those isolated from lake sediment, *Halorubrum lacusprofundi*, or Permian salt mine, *Haloterrigena salifodinae* BOL5-1, *Natrinema versiforme* BOL5-4, and *N. pallidum* BOL6-1. Genomic sequence analysis of the photolyase genes and comparison of their predicted protein sequences and structures indicate that the strains isolated at the surface have conserved key photolyase residues known to be important for function. 

The most UV-tolerant strains, including *Halobacterium* sp. NRC-1, present a resource for further study in order to better understand the mechanisms of UV survival on Earth, and the potential for survival on other planets, including Mars, where a protective ozone layer is not present to shield life from DNA damage. This study adds to the knowledgebase on the functioning of enzymes at the extreme limits to life which promise to be a valuable future resource for biotechnology.

## Figures and Tables

**Table 1:** Microorganisms used in the study.

Haloarchaea	Source	Genome Size (bp)	GC%	Ref.
*Halobacterium* sp. NRC-1	San Francisco Bay, USA	2,571,010	65.9	[27]
*Halobacterium* sp. BOL4-2	Salar de Uyuni, Bolivia	2,428,492	66.3	[38]
*Halobacterium* sp. GSL-19	Great Salt Lake, USA	2,326,224	66.7	[35]
*Salarchaeum* sp. JOR-1	Dead Sea, Jordan	2,523,371	66.2	[36]
*Halorubrum lacusprofundi*	Deep Lake, Antarctica	3,692,576	64.0	[30]
*Halorubrum* sp. BOL3-1	Salar de Uyuni, Bolivia	3,654,453	65.9	[37]
*Haloterrigena salifodinae* BOL5-1	Tarija mine, Bolivia	5,087,240	63.4	[39]
*Natrinema pallidum* BOL6-1	Tarija mine, Bolivia	3,778,093	64.3	[40]
*Natrinema versiforme* BOL5-4	Tarija mine, Bolivia	4,674,473	63.4	[40]

**Table 2:** Key amino acid residues of *S*. *elongatus* PCC 6301 photolyase compared to Phr2 proteins of diverse Haloarchaea.

SEL AA *	Interaction with	SEL	NRC-1 ^†^	JOR-1	Hla	BOL3-1	BOL5-1	BOL6-1
50	HDF	R	R	R	R	R	V	R
139	DNA	S	T	T	T	T	T	P
144	DNA	P	P	P	P	P	H	H
147	DNA	V	V	V	V	V	D	V
148	DNA	Y	Y	Y	Y	Y	F	F
149	DNA	G	T	T	S	T	S	S
150	DNA	P	Y	Y	Y	Y	D	Y
242	FAD	L	L	V	M	L	L	L
279	FAD	W	F	F	Y	F	F	F
282	DNA	E	Q	Q	Q	Q	R	Q
384	FAD	A	N	N	N	N	H	N
385	FAD	N	D	D	D	N	A	D
398	DNA	D	D	D	D	D	N	D
413	DNA	K	R	D	R	R	T	E
460	DNA	Q	R	R	R	R	H	R

* SEL AA (amino acid) residue numbers are used to reference corresponding haloarchaeal photolyase residues in the text. ^†^ NRC-1 residues are the same as BOL4-2 and GSL-19.

## Data Availability

Protein sequences are available in NCBI, and the DNA photolyase structure is available in PDB.
